# Hypoxia-inducible factors enhance glutamate signaling in cancer cells

**DOI:** 10.18632/oncotarget.2593

**Published:** 2014-10-15

**Authors:** Hongxia Hu, Naoharu Takano, Lisha Xiang, Daniele M. Gilkes, Weibo Luo, Gregg L. Semenza

**Affiliations:** ^1^ Predoctoral Training Program in Human Genetics, Johns Hopkins University School of Medicine, Baltimore, MD; ^2^ McKusick-Nathans Institute of Genetic Medicine, Johns Hopkins University School of Medicine, Baltimore, MD; ^3^ Vascular Program, Institute for Cell Engineering, Johns Hopkins University School of Medicine, Baltimore, MD; ^4^ Department of Oncology and Southwest Cancer Center, Southwest Hospital, Third Military Medical University, Chongqing, China; ^5^ Department of Biological Chemistry, Johns Hopkins University School of Medicine, Baltimore, MD; ^6^ Departments of Medicine, Oncology, Pediatrics, Radiation Oncology, Johns Hopkins University School of Medicine, Baltimore, MD

**Keywords:** AMPA receptor, clear cell renal carcinoma, hepatocellular carcinoma, HIF-1

## Abstract

Signaling through glutamate receptors has been reported in human cancers, but the molecular mechanisms are not fully delineated. We report that in hepatocellular carcinoma and clear cell renal carcinoma cells, increased activity of hypoxia-inducible factors (HIFs) due to hypoxia or VHL loss-of-function, respectively, augmented release of glutamate, which was mediated by HIF-dependent expression of the *SLC1A1* and *SLC1A3* genes encoding glutamate transporters. In addition, HIFs coordinately regulated expression of the *GRIA2* and *GRIA3* genes, which encode glutamate receptors. Binding of glutamate to its receptors activated SRC family kinases and downstream pathways, which stimulated cancer cell proliferation, apoptosis resistance, migration and invasion in different cancer cell lines. Thus, coordinate regulation of glutamate transporters and receptors by HIFs was sufficient to activate key signal transduction pathways that promote cancer progression.

## INTRODUCTION

Hypoxia-inducible factors (HIFs) are transcription factors that mediate adaptive responses to reduced oxygen availability. HIFs are heterodimers, composed of an oxygen-regulated HIF-1α or HIF-2α subunit and a constitutively expressed HIF-1β subunit, that bind to the consensus DNA sequence 5′-RCGTG-3′, which is embedded within hypoxia response elements (HREs) in target genes [1]. Proline residues Pro^402^ and Pro^564^ of human HIF-1α (Pro^405^ and Pro^531^ of HIF-2α) are subjected to O_2_ dependent hydroxylation by prolyl hydroxylase domain proteins (PHDs) [2-4]. This modification is required for interaction with the von Hippel-Lindau tumor suppressor protein (VHL), which is the substrate-specific component of an E3 ubiquitin ligase that targets hydroxylated HIF-1α or HIF-2α for ubiquitination and subsequent proteasomal degradation [4].

HIFs have been implicated in many developmental, physiological and pathophysiological processes [3, 5, 6]. HIFs play key roles in many critical aspects of cancer biology, including metabolic reprogramming, proliferation, survival, stem cell maintenance, angiogenesis, epithelial-mesenchymal transition, immune evasion, invasion, metastasis, and resistance to chemotherapy and radiation therapy [7]. Regions of intratumoral hypoxia are present in many solid cancers, leading to induction of HIF activity [8]. In addition, oncogene gain-of-function or tumor suppressor gene loss-of-function also stimulates increased HIF activity in cancer [9]. The most notable example of a genetic alteration driving HIF activity is in clear cell renal cell carcinoma (ccRCC), which has a high incidence of VHL loss-of-function due to either mutation or epigenetic silencing, which leads to high HIF activity even under non-hypoxic conditions [10]. High HIF-1α or HIF-2α abundance in the diagnostic biopsy is associated with metastasis, treatment failure, and patient mortality in many types of cancer [7, 9].

Glutamate is the primary excitatory neurotransmitter of the central nervous system (CNS). However, glutamate signaling has been implicated in various non-excitable tissues, as well as in several diseases including cancer [11]. In glioma, glutamate secretion by cancer cells induces excitotoxic death of neighboring neurons, thereby facilitating tumor growth and invasion [12, 13]. Glutamate antagonists inhibit the proliferation and migration of glioma and other cancer cell types [14]. Glutamate acts on metabotropic or ionotropic cell surface receptors. The metabotropic receptors are G protein-coupled receptors that are encoded by the *GRM1-8* genes. The ionotropic receptors mainly include three subclasses: *N*-methyl-D-aspartate (NMDA) receptors, which are encoded by the *GRIN1*, *GRIN2A-D*, and *GRIN3A-B* genes; α-amino-3-hydroxy-5-methyl-4-isoxazolepropionic acid (AMPA) receptors, which are encoded by the *GRIA1-4* genes; and kainate receptors, which are encoded by the *GRIK1-5* genes [15].

Glutamate receptors have been implicated in several different types of cancer. Insertional mutagenesis of *Grm1* or melanocyte-specific overexpression of *Grm1* or *Grm5* leads to melanoma in transgenic mouse models [16, 17]. The expression of all 24 genes encoding glutamate receptor subunits has been detected at the mRNA level in cancer cell lines [18]. Molecular and biochemical studies of glutamate receptors have demonstrated their roles in various cancer types [19-22]. High-throughput genomic studies have identified *GRM1*, *GRM3*, *GRM4*, *GRM8* and *GRIN2A* as susceptibility genes in non-small-cell lung cancer (NSCLC), melanoma, osteosarcoma, and bladder cancer [23-27]. In contrast, *GRIK2*, which is the gene most frequently involved in chromosome 6q deletions in acute lymphocytic leukemia, is regarded as a tumor suppressor gene [28]. Additionally, hypermethylation of *GRIK1*, *GRIK2*, *GRIN2A* and *GRIN2B* has been reported in ccRCC, gastric cancer, colon cancer, esophageal squamous cell carcinoma and NSCLC [29-34]. Thus, the effect of gain or loss of glutamate receptor function varies in different cancers.

In the present study, we demonstrated that HIF activity, induced by hypoxia or VHL loss-of-function in hepatocellular and renal carcinoma cells, respectively, mediated the coordinate transcription of multiple genes encoding glutamate transporters and glutamate receptors, which resulted in activation of signal transduction pathways that stimulated cancer cell proliferation, survival, or migration and invasion. Our results demonstrate that HIFs mediate glutamate signaling that promotes cancer progression.

## RESULTS

### Hypoxia induces glutamate release and the expression of genes encoding glutamate transporters in Hep3B cells

**Figure 1:**
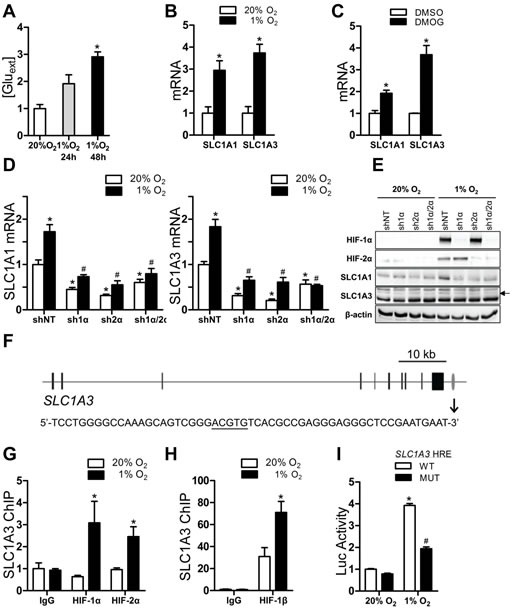
Glutamate release and transporter expression in Hep3B cells (A) Cells were cultured for indicated time and glutamate concentration in medium was determined and normalized to 20% O_2_. ^*^P < 0.05 vs 20% O_2_, one-way ANOVA with Dunnett post-test. (B-C) Cells were exposed to 20% or 1% O_2_ (B), or to vehicle (DMSO) or DMOG (C) for 24 h. mRNAs were analyzed by RT-qPCR and normalized to 20% O_2_ or DMSO.^*^P < 0.05 vs 20% O_2_ or DMSO, Student's *t* test. (D) mRNAs were analyzed in subclones expressing shRNA directed against HIF-1α, HIF-2α or both that were exposed to 20% or 1% O_2_ for 24 h. ^*^P < 0.05 vs shNT at 20% O_2_; ^#^*P* < 0.05 vs shNT at 1% O_2_; two-way ANOVA/Bonferroni post-test. (E) Immunoblot assays were performed using lysates from subclones exposed to 20% or 1% O_2_; arrow indicates the SLC1A3-specific band. (F) *SLC1A3* exons and HRE are indicated by black bars and grey oval, respectively. HRE nucleotide sequence is shown below. (G-H) Cells were exposed to 20% or 1% O_2_ for 24 h. ChIP assays were performed using IgG or indicated antibody. ^*^P < 0.05 vs 20% O_2_, ANOVA with Bonferroni post-test. (I) Luciferase (Luc) activity was determined in cells co-transfected with pSV-Renilla and a firefly luciferase reporter containing the wild-type (WT) or mutant *SLC1A3* hypoxia response element (HRE). ^*^P < 0.05 vs WT at 20% O_2_, ^#^*P* < 0.05 vs WT at 1% O_2_, ANOVA with Bonferroni post-test. Data are mean ± SEM or a representative blot from ≥ 3 experiments.

Human glioma, mouse melanoma, rat prostate cancer, and human breast cancer cells have been shown to release glutamate [12, 35]. Because high concentrations of extracellular glutamate also accumulate in response to cerebral ischemia [36], we hypothesized that hypoxia may induce glutamate release from cancer cells. To test this, we maintained human hepatocellular carcinoma Hep3B cells at 20% O_2_ or exposed the cells to 1% O_2_ for 24 or 48 h. We observed a time-dependent increase of extracellular glutamate in the media of cells exposed to hypoxia, as compared to cells maintained at 20% O_2_ (Fig. 1A), indicating that reduced oxygen availability triggers increased glutamate release from Hep3B cells.

There are several molecular mechanisms by which glutamate release is mediated: vesicular glutamate transporters (encoded by genes *SLC17A6-8*); the cystine-glutamate antiporter system x_c_^−^ (encoded by *SLC7A11* and *SLC3A2*); and the membrane glutamate transporters EAAT3, EAAT2, EAAT1, EAAT4, and EAAT5 (encoded by *SLC1A1*, *SLC1A2*, *SLC1A3*, *SLC1A6*, and *SLC1A7*). Reverse transcription and quantitative real-time PCR (RT-qPCR) analysis of Hep3B cells exposed to 20% or 1% O_2_ for 24 h revealed that the abundance of *SLC1A1* and *SLC1A3* mRNA, but not that of mRNAs encoding other glutamate transporters, was significantly induced by hypoxia (Fig. 1B and Fig. S1A). Hypoxia did not induce *SLC1A1* and *SLC1A3* mRNA in two breast cancer cell lines (Fig. S1B-C). *SLC1A1* and *SLC1A3* mRNA expression was also increased when Hep3B cells were treated with 100 μM dimethyloxalylglycine (DMOG), which inhibits PHD activity (Fig. 1C).

### HIFs mediate SLC1A1 and SLC1A3 gene expression in hypoxic Hep3B cells

To determine whether HIF-1 or HIF-2 was directly responsible for the hypoxia-induced expression of *SLC1A1* and *SLC1A3*, Hep3B cells were stably transfected with vectors encoding: a short hairpin RNA (shRNA) that was non-targeting (shNT); shRNA targeting HIF-1α (sh1α) or HIF-2α (sh2α); or shRNAs targeting both HIF-1α and HIF-2α (sh1α/2α). Analysis of SLC1A1 and SLC1A3 mRNA (Fig. 1D) and protein (Fig.1E) revealed that hypoxia-induced expression was abrogated when HIF-1α, HIF-2α, or both were knocked down in Hep3B cells. The effects of these shRNAs on their targets were confirmed by immunoblot assays (Fig.1E).

To examine whether *SLC1A3* was a direct HIF target gene, we performed chromatin immunoprecipitation (ChIP) assays with primers flanking HIF consensus binding site sequences along the gene. One site located 2 kb downstream of the *SLC1A3* gene (gray oval in Fig. 1F, top) was enriched by immunoprecipitation of chromatin from hypoxic cells with HIF-1α, HIF-2α (Fig. 1G), or HIF-1β (Fig. 1H) antibody. To test whether this HIF-binding site was embedded in an HRE, a 55-bp wild-type (WT) sequence spanning the site (Fig. 1F, bottom, HIF binding site is underscored) was inserted into the firefly luciferase reporter plasmid pGL2-promoter. Hep3B cells co-transfected with this *SLC1A3* HRE reporter and a control pSV-Renilla luciferase reporter were exposed to 20% or 1% O_2_. The ratio of firefly:Renilla luciferase activity increased with hypoxic exposure. Mutation of the HIF binding site in the *SLC1A3* HRE (5′-ACGTG-3′ to 5′-AAAAG-3′) significantly impaired hypoxia-induced luciferase activity (Fig. 1I). Taken together, these results suggested that HIF-dependent increases in SLC1A1 and SLC1A3 expression may contribute to increased efflux of glutamate from Hep3B cells under hypoxic conditions.

### Glutamate receptor signaling stimulates proliferation of hypoxic Hep3B cells

**Figure 2:**
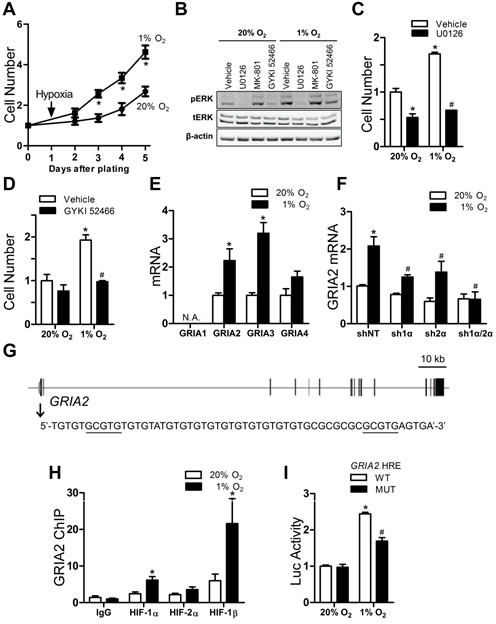
Glutamate receptor signaling and proliferation of Hep3B cells (A) Cells were exposed to 20% or 1% O_2_ and counted. ^*^P < 0.05 vs 20% O_2_, ANOVA with Bonferroni post-test. (B) Immunoblot analysis was performed on cells exposed to 20% or 1% O_2_ + vehicle, U0126, MK-801, or GYKI 52466 for 24 h. (C-D) Cells were exposed to 20% or 1% O_2_ + vehicle or either GYKI 52466 (C) or U0126 (D) for 96 h and counted. ^*^P < 0.05 vs vehicle-20% O_2_, ^#^*P* < 0.05 vs vehicle-1% O_2_, ANOVA with Bonferroni post-test. (E) Cells were exposed to 20% or 1% O_2_ for 24 h and mRNAs were analyzed. ^*^P < 0.05 vs 20%, Student's *t* test. (F) *GRIA2* mRNA abundance was analyzed in shRNA-expressing subclones exposed to 20% or 1% O_2_ for 24 h. ^*^P < 0.05 vs shNT-20% O_2_; ^#^*P* < 0.05 vs shNT-1% O_2_, two-way ANOVA with Bonferroni post-test. (G) *GRIA2* exons and HRE are indicated by black bars and grey oval, respectively; HRE sequence is shown below. (H) ChIP assays of cells that were exposed to 20% or 1% O_2_ for 24 h. ^*^P < 0.05 vs 20%, ANOVA with Bonferroni post-test. (I) Luciferase (Luc) activity was determined in cells co-transfected with pSV-Renilla and a firefly luciferase reporter containing the wild-type or mutant *SLC1A3* HRE. ^*^P < 0.05 vs WT-20% O_2_, ^#^*P* < 0.05 vs WT-1% O_2_, ANOVA with Bonferroni post-test. Data are mean ± SEM or a representative blot from ≥ 3 experiments.

Whereas the proliferation of most cells is inhibited by hypoxia, the proliferation of Hep3B cells was significantly increased under hypoxic conditions (Fig. 2A). Since the mitogen-activated protein kinase (MAPK) signaling cascade leading to the phosphorylation/activation of ERK1/2 is a major pathway for extracellular signal-induced cell proliferation, we examined the phosphorylation of ERK1/2 in cells exposed to vehicle or antagonists of various classes of glutamate receptors, including the NMDA receptor antagonist MK-801 and the AMPA receptor antagonist GYKI 52466. Phosphorylation of ERK1/2 was increased in vehicle-treated cells exposed to 1% O_2_. Hypoxia-induced ERK1/2 phosphorylation was inhibited by GYKI 52466, but not by MK-801. ERK1/2 is activated by the upstream MAPK/ERK kinase (MEK) and the MEK inhibitor U0126 completely abolished phosphorylation of ERK1/2 (Fig. 2B) and inhibited cell proliferation under both 20% and 1% O_2_ (Fig. 2C). In contrast, incubation of Hep3B cells with GYKI 52466 inhibited phosphorylation of ERK1/2 and cell proliferation specifically under 1% O_2_ (Fig. 2D). Although MK-801 had no effect on hypoxia-induced ERK1/2 phosphorylation, it did reduce cell proliferation (Fig. S2A), which is consistent with a previous report that MK-801 blocks growth of hepatocellular carcinoma cells by increasing FOXO activity [37]. Taken together, these results suggest that glutamate released by hypoxic Hep3B cells binds to AMPA receptors and stimulates MEK-ERK signaling, leading to increased proliferation.

### HIF-1 mediates hypoxia-induced expression of glutamate receptors

AMPA receptors are homotetramers or heterotetramers composed of subunits GluR1 to GluR4, which are encoded by *GRIA1* to *GRIA4*. AMPA receptors are responsible for the vast majority of fast excitatory synaptic transmission within the mammalian central nervous system [38]. Expression of *GRIA2* and *GRIA3* mRNA was induced by hypoxia in Hep3B cells (Fig. 2E) but not in two breast cancer cell lines (Fig. S2B-C). Increased GRIA2 and GRIA3 protein expression was also induced by hypoxia (Fig. S2D). Hypoxic induction of *GRIA2* and *GRIA3* mRNA expression was significantly inhibited when expression of HIF-1α or HIF-2α, and especially when expression of both HIF-1α and HIF-2α, was knocked down in Hep3B cells (Fig. 2F and Fig. S2E). ChIP assays revealed that a DNA sequence encompassing two copies of 5′-GCGTG-3′, with one copy at −26 nt and the other copy at +15 nt relative to the *GRIA2* transcription start site (Fig. 2G), was enriched by immunoprecipitation of chromatin from hypoxic Hep3B cells with HIF-1α or HIF-1β antibodies (Fig. 2H). A 55-bp WT sequence (Fig. 2G, bottom) spanning the HIF binding sites, or the same sequence with mutation of 5′-GCGTG-3′ to 5′-GAAAG-3′ (MUT), was inserted into pGL2-promoter. Hypoxia increased the ratio of firefly:Renilla luciferase to a greater extent in Hep3B cells coexpressing the *GRIA2* HRE-WT reporter and pSV-Renilla compared to cells transfected with HRE-MUT (Figure 2I). Taken together, these results indicate that expression of *GRIA2* and *GRIA3* is induced by hypoxia in a HIF-dependent manner and that, at least in the case of *GRIA2*, this results from direct binding of HIF-1 to the target gene.

### FYN is a HIF-regulated functional intermediary between GRIA2/3 and ERK1/2

**Figure 3:**
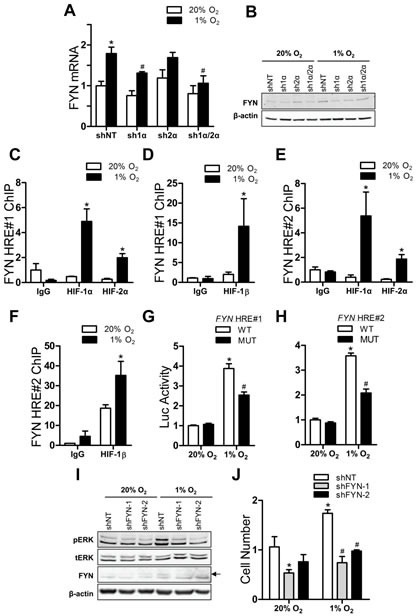
HIF-dependent FYN signaling to ERK in hypoxic Hep3B cells (A) FYN mRNA levels were analyzed in shRNA-expressing subclones exposed to 20% or 1% O_2_. ^*^P < 0.05 vs shNT-20% O_2_, ^#^*P* < 0.05 vs shNT-1% O_2_, two-way ANOVA with Bonferroni post-test. (B) Immunoblot analysis was performed on shRNA-expressing subclones cultured at 20% or 1% O_2_. (C-F) ChIP assays were performed in cells exposed to 20% or 1% O_2_ using primers flanking *FYN* HRE#1 (C-D) or primers flanking *FYN* HRE#2 (E-F). ^*^P < 0.05 vs 20% O_2_, ANOVA with Bonferroni post-test. (G-H) Luciferase activity was determined in cells expressing a reporter containing HRE#1 (G) or HRE#2 (H). ^*^P < 0.05 vs WT-20% O_2_, #*P* < 0.05 vs WT-1% O_2_, ANOVA with Bonferroni post-test. (I) Immunoblot assays of control (shNT) and FYN knockdown (shFYN-1 and shFYN-2) subclones exposed to 20% or 1% O_2_ for 24 h; arrow indicates FYN-specific band. (J) Cells were incubated in 20% or 1% O_2_ for 72 h and counted. ^*^P < 0.05 vs shNT-20% O_2_, ^#^*P* < 0.05 vs shNT-1% O_2_, ANOVA with Bonferroni post-test. Data are mean ± SEM or a representative blot from ≥ 3 experiments.

The Src family tyrosine kinase LYN physically and functionally associates with AMPA receptors and is involved in the AMPA-receptor-mediated activation of MAPK signaling in primary cell cultures from the cerebellum [39]. Expression of another Src family member, FYN, is upregulated in ccRCC cell lines with HIF activation [40]. We investigated whether FYN expression was regulated by HIFs in Hep3B cells and whether it transduced signals from AMPA receptors to ERK. FYN mRNA (Fig. 3A) and protein (Fig. 3B) abundance was increased by hypoxia in the Hep3B subclone expressing a non-targeted control shRNA (shNT), an increase that was abrogated in the HIF-1α knockdown (sh1α) and the HIF-1α and HIF-2α double knockdown (sh1α/2α) subclones, but not in the HIF-2α knockdown (sh2α) subclone. In contrast, the abundance of other SRC family kinases including SRC, LYN, and LCK was not affected by hypoxia (Fig. S3A). FYN mRNA expression was not induced by hypoxia in two breast cancer cell lines (Fig. S3B). Introns 2 and 13 of the *FYN* gene each contained a candidate HRE (designated HRE#1 and HRE#2, respectively) (Fig. S3C). ChIP assays revealed that hypoxia induced significant binding of HIF-1α, HIF-2α (Fig. 3C and 3E) and HIF-1β (Fig. 3D and 3F) to both sites. A 55-bp sequence spanning either wild type (WT) or mutant (MUT; 5′-ACGTG-3′ to 5′-AAAAG-3′) HIF binding site was inserted into pGL2-promoter. Firefly luciferase activity was significantly induced by hypoxia in Hep3B cells transfected with *FYN* HRE#1-WT (Fig. 3G) or *FYN* HRE#2-WT (Fig. 3H), and the hypoxic induction was significantly decreased in cells transfected with HRE#1-MUT or HRE#2-MUT.

To confirm that FYN signals to ERK1/2, we stably knocked down FYN expression in Hep3B cells with two different shRNAs, shFYN-1 and shFYN-2 (Fig. S3D and Fig. 3I). ERK phosphorylation was induced by hypoxia in the shNT subclone, but not in the shFYN-1 or shFYN-2 subclone (Fig. 3I). In addition, hypoxia significantly increased proliferation of the shNT subclone but not the shFYN subclones (Fig. 3J). Taken together, these results indicate that FYN is a HIF-regulated intermediary that is required for the activation of ERK1/2 signaling and proliferation of Hep3B cells exposed to hypoxia.

### AMPA receptor antagonist inhibits Hep3B tumor xenograft growth

**Figure 4:**
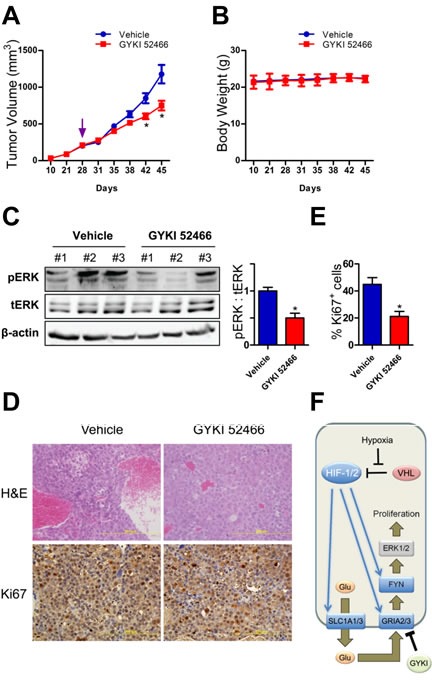
Glutamate receptor signaling in Hep3B tumor xenografts (A-B) Mice with xenografts that reached 200 mm^3^ were treated with vehicle or GYKI 52466. Tumor volume (A) and body weight (B) were monitored. ^*^P < 0.05 vs vehicle, ANOVA/Bonferroni post-test (mean ± SEM; n = 3 mice per treatment). (C) Tumor lysates were subjected to immunoblot assays (left) and the ratio of phosphorylated:total ERK (pERK/tERK) was determined (right).^*^P < 0.05 vs vehicle, Student's *t* test (mean ± SEM; n = 3 mice per treatment). (D) Tumor sections were stained with hematoxylin and eosin (upper panels) and Ki67 (lower panels). (E) The percentage of cells positively stained with Ki67 was quantified. ^*^P < 0.05 vs vehicle, Student's *t* test (mean ± SEM; n = 3 mice per treatment). (F) The mechanisms and consequences of HIF-dependent glutamate signaling in hypoxic Hep3B cells are shown.

Having demonstrated that activation of AMPA-type glutamate receptors stimulates FYN → ERK signaling that results in the proliferation of hypoxic Hep3B cells, we next asked whether inhibiting this pathway using the AMPA receptor antagonist GYKI 52466 would have anti-cancer effects *in vivo*. Hep3B cells were injected subcutaneously into the right flank of severe combined immune deficiency (SCID) mice. Administration of the AMPA receptor antagonist significantly impaired tumor growth (Fig. 4A) without affecting body weight (Fig. 4B). Immunoblot analysis revealed that tumors from GYKI 52466 treated mice had a significantly decreased ratio of phosphorylated:total ERK (Fig. 4C). Ki67 immunohistochemistry, as a measure of cancer cell proliferation *in vivo*, revealed a significant decrease in the number of Ki67^+^ cells in xenografts from mice treated with GYKI 52466 (Fig. 4D and Fig. 4E). Taken together, the data in Fig. 1-4 indicate that a signaling pathway consisting of HIFs, SLC1A1/3, GRIA2/3, FYN, and ERK1/2 contributes to cell proliferation in Hep3B cells cultured under hypoxic conditions and in Hep3B tumor xenografts (Fig. 4F).

We next analyzed the prognostic implication of the HIF regulated genes involved in glutamate signaling in hepatocellular carcinoma (*SLC1A1*, *SLC1A3*, *GRIA2*, *GRIA3* and *FYN*) using PROGgene [41] to analyze the GSE 10141 mRNA expression data set from 80 surgically resected hepatomas [42]. High expression of the gene set (greater than the median value) was significantly associated with decreased patient survival (hazard ratio = 2.7, *P* < 0.05; Fig. S3E).

### SLC1A1/3, GRIA3, and FYN expression correlate with HIF target genes in ccRCC

Next, we investigated whether the glutamate-AMPA receptor-SRC family kinase signaling axis is active in other cancer cell types. Given the key role of HIFs in this pathway, we analyzed ccRCCs because VHL loss-of-function is common and represents an O_2_-independent mechanism to activate HIFs in cancer cells. Analyzing gene expression data from 42 ccRCCs in the International Cancer Genome Consortium [43] revealed that SLC1A1, GRIA3 and FYN mRNA abundance was each significantly correlated with the expression of six known HIF target genes (Table 1). SLC1A3 mRNA abundance was significantly correlated with 3 of the 6 target genes. For comparison, expression of *PKM2*, another HIF target gene, was also correlated with 3 of the 6 genes. *RPL4* was analyzed as a representative non-HIF target gene. As expected, none of the above genes showed significant correlation with *RPL4*. These data suggested that genes involved in glutamate receptor signaling might be regulated by HIFs in ccRCC.

**Table 1:** *SLC1A1*, *SLC1A3*, *GRIA3* and *FYN* gene expression is correlated with the expression of known HIF target genes in clear cell renal cell carcinomas

Category	GENE		*SLC1A1*	*SLC1A3*	*GRIA3*	*FYN*	*PKM2*
HIF Targets		*SLC2A1*	*	*	*	***	*
		*LDHA*	***	*n.s.*	**	***	*n.s.*
		*BNIP3*	***	*n.s.*	**	***	*n.s.*
		*CA9*	***	**	**	***	*
		*SLC16A3*	***	**	***	***	*
		*VEGFA*	***	*n.s.*	***	***	*n.s*.
Non-HIF target		*RPL4*	*n.s.*	*n.s.*	*n.s.*	*n.s.*	*n.s.*

* *P* < 0.05,

** *P* < 0.01,

*** *P* < 0.001, *n.s*., not significant, Pearson's correlation test of microarray data from The Cancer Genome Atlas (TCGA) dataset of 42 renal clear cell carcinomas.

### HIF-dependent glutamate signaling stimulates survival of 786-O cells

**Figure 5:**
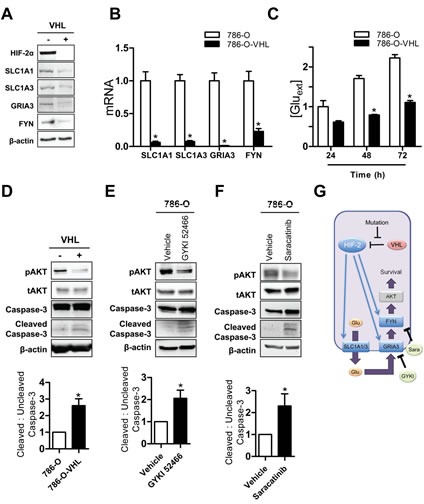
HIF-mediated glutamate signaling in 786-O ccRCC cells (A-B) Immunoblot (A) and RT-qPCR (B) assays of 786-O (-) and 786-O-VHL (+) cells. ^*^P < 0.05 vs 786-O. (C) Glutamate concentrations in media were measured, corrected for cell number, and normalized to results for 786-O cells cultured for 24 h. ^*^P < 0.05 vs 786-O at 48 h and at 72 h, Student's *t* test. (D) Immunoblot assays were performed to detect: phosphorylated AKT (pAKT-Ser473) and total AKT (tAKT); and full length uncleaved and cleaved Caspase-3. (E-F) 786-O cells were treated with vehicle, GYKI 52466 (E), or Saracatinib (F) for 24 h and cell lysates were subjected to immunoblot assays. Quantification of the ratio of the cleaved Caspase 3 relative to the full length uncleaved Caspase-3 in the experiments shown in panel D, E and F below the blots. ^*^P < 0.05, Student's *t* test. (G) The mechanisms and consequences of HIF-mediated glutamate signaling in 786-O cells are shown. Data are mean ± SEM or a representative blot from ≥ 3 experiments.

Because one-third of ccRCC tumors with VHL loss-of-function exclusively overexpress HIF-2α (“H2”) and two-thirds overexpress both HIF-1α and HIF-2α (“H1H2”) [44], we analyzed two *VHL* mutant ccRCC cell lines, 786-O and RCC4, which represent the H2 and H1H2 subtypes, respectively. Only HIF-2α was constitutively expressed in 786-O cells and, after reintroduction of wild-type VHL, HIF-2α expression was markedly decreased under non-hypoxic conditions (Fig. 5A). A decrease in SLC1A1, SLC1A3, GRIA3 and FYN protein and mRNA abundance was observed in the 786-O-VHL subclone (Fig. 5A-B). Extracellular glutamate accumulated in a time-dependent manner in the media of 786-O and 786-O-VHL cells. However, conditioned medium from 786-O cells contained significantly more glutamate than 786-O-VHL at every time point (Fig. 5C). We examined the effects of Evans Blue, an inhibitor of vesicular glutamate transporters, and Sulfasalazine, an inhibitor of the cystine-glutamate antiporter [45], on extracellular glutamate concentrations. Neither inhibitor affected cell proliferation (Fig. S4A) or extracellular glutamate concentrations (Fig. S4B). These results suggest that glutamate efflux in 786-O cells is mediated by the membrane glutamate transporters SLC1A1 and SLC1A3.

In contrast to the effect of HIF activation in Hep3B cells, 786-O cells did not have any growth advantage over 786-O-VHL cells (Fig. S4A). However, 786-O cells exhibited decreased apoptosis compared to 786-O-VHL cells, as measured by Annexin V staining and flow cytometry (Fig. S5). The PI3K/AKT pathway is a major anti-apoptotic signaling pathway and AKT phosphorylation (at Ser-473) was increased, and cleaved (activated) caspase 3 was decreased, in 786-O as compared to 786-O-VHL cells (Fig. 5D). To determine whether glutamate-glutamate receptor-SRC family kinase signaling was involved, we treated 786-O cells with either the AMPA receptor antagonist GYKI 52466 or the SRC family kinase inhibitor Saracatinib. Both inhibitors decreased the phosphorylation of AKT and increased caspase-3 cleavage (Fig. 5E and 5F). Taken together, these data indicate that in 786-O ccRCC cells, HIF-2 → SLC1A1/3 → GRIA3 → FYN → AKT signaling stimulates cell survival by inhibiting apoptosis.

### HIF-dependent glutamate signaling stimulates RCC4 migration and invasion

**Figure 6:**
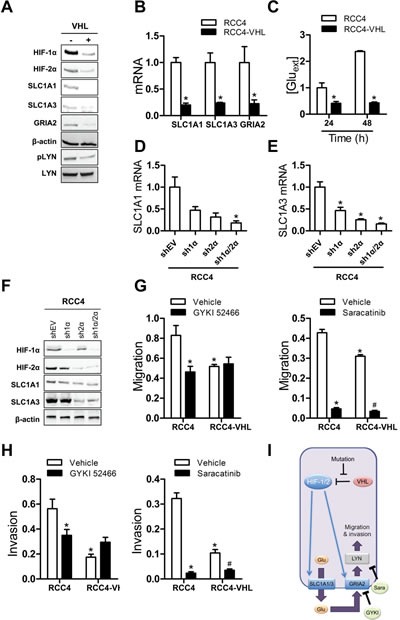
HIF-mediated glutamate signaling in RCC4 cells (A-B) Immunoblot (A) and RT-qPCR (B) assays of RCC4 (-) and RCC4-VHL (+) cells were performed. ^*^P < 0.05 vs RCC4, Student's *t* test. (C) Glutamate concentrations were measured in conditioned media, and normalized to RCC4 at 24 h.^*^P < 0.05 vs RCC4 at 24 h and at 48 h, Student's *t* test. (D-F) RT-qPCR (D, E) and immunoblot (F) assays were performed with RCC4 subclones. ^*^P < 0.05 vs shEV, ANOVA with Bonferroni post-test. (G) Cells were seeded in a Boyden chamber in the presence of vehicle, GYKI 52466 (left panel), or Saracatinib (right panel) and migration through uncoated inserts in response to FBS was determined by crystal violet staining. ^*^P < 0.05 vs RCC4-vehicle, ^#^*P* < 0.05 vs RCC4-VHL-vehicle. (H) Cells were seeded on Matrigel-coated inserts in the presence of vehicle, GYKI 52466 (left panel), or 10 μM Saracatinib (right panel). Cells that invaded through the coated inserts were determined by crystal violet staining. ^*^P < 0.05 vs RCC4-vehicle; ^#^*P* < 0.05 vs RCC4-VHL-vehicle. (I) The mechanisms and consequences of HIF-mediated glutamate signaling in RCC4 cells are shown. Data are mean ± SEM or a representative blot from ≥ 3 experiments.

To further confirm the involvement of glutamate signaling in ccRCC cells, we analyzed RCC4 cells, which are VHL-null cells that constitutively express both HIF-1α and HIF-2α protein under non-hypoxic conditions. Stable transfection of an expression vector encoding wild-type VHL abolished the increased abundance of HIF-1α and HIF-2α protein under non-hypoxic conditions (Fig. 6A). Similarly, the abundance of glutamate transporters SLC1A1 and SLC1A3 was decreased at the protein (Fig. 6A) and mRNA (Fig. 6B) levels in the RCC4-VHL subclone. RCC4 cells responded differently than 786-O cells to re-expression of wild type VHL. First, GRIA2 protein levels were reduced; and second, the abundance of FYN was not affected, but the phosphorylation of LYN, another SRC family kinase member, was decreased in RCC4-VHL cells (Fig. 6A-B). However, as in the case of 786-O, VHL-null RCC4 cells released significantly more glutamate than the RCC4-VHL subclone (Fig. 6C). In addition, when RCC4-VHL cells were exposed to hypoxia, these cells released more glutamate (Fig. S6).

We next analyzed RCC4 subclones that stably expressed an empty vector (shEV) or vector encoding HIF-1α (sh1α) or HIF-2α (sh2α) shRNA, or vectors encoding both HIF-1α and HIF-2α shRNAs (sh1α/2α) to determine whether HIF-1α or HIF-2α was specifically required for *SLC1A1* and *SLC1A3* expression in RCC4 cells. SLC1A1 and SLC1A3 mRNA (Fig. 6D-E) and protein (Fig. 6F) overexpression were abrogated when HIF-1α, HIF-2α, or both were knocked down in RCC4 cells.

In contrast to the increased proliferation of Hep3B cells under hypoxia and the decreased apoptosis of VHL-null 786-O cells, we found that VHL-null RCC4 cells exhibited increased migration and invasion as compared to RCC4-VHL cells (Fig. 6G-H, left panel). The glutamate receptor antagonist GYKI 52466 significantly impaired migration and invasion in RCC4 cells but not RCC4-VHL cells (Fig. 6G-H, left panel). In contrast, Saracatinib decreased migration and invasion in both RCC4 and RCC4-VHL cells (Fig. 6G-H, right panel), demonstrating that SRC family kinase activity is required for basal as well as HIF-dependent migration and invasion. Taken together, the data indicate that HIF-1/2 → SLC1A1/3 → GRIA2 → LYN signaling stimulates migration and invasion of VHL-null RCC4 cells (Fig. 6I).

## DISCUSSION

The results presented here delineate molecular mechanisms by which HIFs mediate enhanced glutamate receptor signaling in Hep3B, 786-O, and RCC4 cancer cells through the direct, coordinate transcriptional activation of multiple genes encoding glutamate transporters and glutamate receptors in response to HIF activity resulting either from hypoxia or VHL loss-of-function. In Hep3B and 786-O cells, HIFs also activated transcription of the *FYN* gene, which served to amplify signaling downstream of the glutamate receptors. Despite the similar HIF-mediated transcriptional responses and activation of SRC family kinases, the ultimate biological consequences of glutamate signaling were different: in Hep3B cells, FYN signaled to ERK1/2 to stimulate cell proliferation; in 786-O cells, FYN signaled to AKT to stimulate cell survival; and in RCC4 cells, LYN signaling stimulated cell migration and invasion.

### Hypoxia induces cancer cell glutamate efflux mediated by SLC1A1 and SLC1A3

Prior studies demonstrated that cancer cells release glutamate, but our data demonstrate that increased glutamate efflux occurs in hepatocellular carcinoma cells in response to hypoxic conditions and in ccRCC cells due to VHL loss-of-function. In both cases, augmented glutamate efflux results from HIF-dependent *SLC1A1* and *SLC1A3* expression. The membrane glutamate transporters encoded by these genes conventionally mediate glutamate transport into cells under physiological conditions, but glutamate transport is reversible, with glutamate efflux occurring in ischemic tissue [46]. Over 50% of glutamate release by glioma cell lines was attributed to the x_c_^−^ cystine/glutamate antiporter, which has been implicated in other cancer cells that release glutamate [45, 47]. However, the reported increase of glutamate and decrease of cystine in the media was not equimolar, with 2- to 7-fold more glutamate secreted [48], which suggests that even under non-hypoxic conditions additional mechanisms must contribute to glutamate release in those cancer cells. SLC1A2, another major membrane glutamate transporter in the CNS, mediates up to 95% of glutamate uptake in the brain and is frequently silenced in gliomas due to DNA methylation [49, 50]. Neither *SLC7A11* nor *SLC1A2* mRNA levels were affected by HIF activation in Hep3B cells or ccRCC cells, and inhibitors of the cystine/glutamate antiporter or vesicular glutamate transporter activity had no effect on extracellular glutamate concentration in 786-O cells, excluding their contribution to HIF-mediated glutamate release in these cells.

### Functional consequences of glutamate receptor signaling vary among cancer cell lines

Several prior studies reported functional effects of AMPA receptors in cancer. *GRIA1* and *GRIA3* were shown to promote tumor progression in glioma [20, 51] and pancreatic cancer [52]. In contrast, *GRIA4* is subject to DNA methylation [53, 54] and inhibition of *GRIA4* expression increased cancer cell proliferation [22]. In the case of *GRIA2*, there are conflicting reports regarding its role in cancer biology. *GRIA2* expression is upregulated in uterine leiomyoma and gastrointestinal neuroendocrine carcinoma compared to adjacent normal tissues [55-57]. In contrast, *GRIA2* expression is lost in high-grade glioma and forced expression in glioma cells inhibited proliferation and induced apoptosis [58]. In pancreatic ductal adenocarcinoma, AMPA receptor signaling to KRAS and MAPK promoted migration and invasion [59]. However, the molecular basis for AMPA receptor expression was not determined. In the current study, we demonstrate that HIF-dependent *GRIA2* and *GRIA3* expression in hepatocellular carcinoma and ccRCC cell lines promotes tumor progression through effects on proliferation, survival, migration and invasion. Analysis of gene expression data from human ccRCC biopsies indicates that the same transcriptional circuits are active *in vivo*, thereby establishing the clinical relevance of our findings.

We have shown that hypoxia is a microenvironmental stimulus that triggers HIF-dependent expression of membrane glutamate transporters as well as AMPA-type glutamate receptors in hepatocellular carcinoma cells. In a mouse model of pancreatic neuroendocrine tumor and in selected human cancers including breast cancer, increased interstitial fluid pressure was reported to stimulate increased expression of vesicular glutamate transporters, activation of NMDA-type glutamate receptors and downstream MEK-MAPK and CaM kinase signaling, leading to proliferation and invasion [60]. Taken together, these results suggest that glutamate signaling is common among human cancers whereas the activating mechanisms are different.

### Therapeutic implications

Our results demonstrate that HIF-mediated glutamate efflux and signaling via AMPA-type glutamate receptors promote multiple aspects of cancer progression and that administration of an AMPA receptor antagonist significantly inhibits Hep3B tumor xenograft growth. The AMPA receptor antagonist talampanel was well tolerated but had no activity as a single agent in a Phase II clinical trial involving patients with recurrent glioma [61]. Previous studies have demonstrated that administration of HIF inhibitors blocks the growth of Hep3B [62] and ccRCC [63] tumor xenografts. In addition to blocking glutamate receptor signaling, HIF inhibitors block many other pathways that are critical for cancer progression [7-9, 64, 65]. Further studies are needed to evaluate the potential benefit of HIF inhibitors in the treatment of advanced liver and kidney cancers, which respond poorly to current chemotherapy regimens.

## METHODS

### Cell culture and reagents

Hep3B, 786-O, RCC4, MDA-MB-231, MDA-MB-435 and HEK293T cells were cultured in Dulbecco's Modified Eagle's Medium (DMEM) supplemented with 10% heat-inactivated fetal bovine serum, 100 units/mL of penicillin, and 100 μg/mL of streptomycin. G418 (0.8 mg/mL) was added to the medium of 786-O-VHL and RCC4-VHL cells. Culture conditions for RCC4-shEV, RCC4-sh1α, RCC4-sh2α, and RCC4-sh1α/2α were as described [66]. Cells were maintained at 37ºC in a 5% CO_2_/95% air incubator. For hypoxic exposure, cells were placed in a modular incubator chamber (Billups-Rothenberg) flushed with 1%O_2_/5% CO_2_/balance N_2_ and incubated at 37ºC. GYKI 52466, MK-801, Evans Blue and Sulfasalazine were purchased from Sigma-Aldrich. U0126 was from LC laboratories. Saracatinib was purchased from Selleckchem.

### Plasmid constructs

The indicated 55-bp oligonucleotides were inserted into pGL2-Promoter (Promega). All plasmid constructs were confirmed by nucleotide sequencing.

### shRNA and lentivirus production

Recombinant lentivirus was generated by transfection of HEK293T cells with the transducing vector pLKO.1-puro encoding shRNA, together with packaging vectors pMD.G and pCMV-dR8.91, using PolyJet (SignaGen). After 48 h, medium containing viral particles was harvested and passed through a 0.45-μm filter (Millipore). Hep3B cells were transduced with viral supernatant in the presence of 8 μg/mL of Polybrene (Sigma-Aldrich). After 24 h, cells were replenished with fresh medium containing 2 μg/mL of puromycin. Cells were maintained in puromycin-containing medium for selection of stable transfectants. shRNA sequences are listed in Table S1.

### Glutamate measurement

Glutamate concentrations in media were measured using a colorimetric glutamate assay (BioVision) in which optical density at λ=450 nm was determined using a microplate reader (PerkinElmer) and glutamate concentration was interpolated from a standard curve and corrected for differences in cell number.

### RT and qPCR

RNA was extracted using TRIzol (Invitrogen), precipitated with isopropanol, treated with DNase I (Ambion), and reverse transcribed with the iScript cDNA Synthesis kit (Bio-Rad). qPCR analysis was performed using Maxima SYBR Green Master Mix (Fermentas) with the iCycler Real-time PCR Detection System (BioRad). The 2^−ΔΔCt^ method was used to calculate relative gene expression. Results were normalized to the 18S rRNA signal. Primer sequences are listed in Table S1.

### Immunoblot assays

Whole cell lysates were prepared in modified RIPA buffer and proteins were fractionated by SDS-PAGE. The following primary antibodies were used: HIF-1α (BD Biosciences); HIF-2α, SLC1A1, SLC1A3, GRIA2, GRIA3, FYN, pLYN (Tyr396), LYN, SRC, LCK, ERK, Caspase-3, and AKT (Novus Biologicals); and phosphorylated ERK, phosphorylated AKT (Ser473), and β-actin (Santa Cruz). HRP-conjugated secondary rabbit antibody was purchased from GE Healthcare Life Sciences; all other secondary antibodies were obtained from Santa Cruz.

### ChIP assays

Hep3B cells were cross-linked with 1% formaldehyde for 10 min and quenched in 0.125 M glycine. Cells were sonicated using a Bioruptor (Diagenode). Sonicated lysates were precleared with salmon sperm DNA/protein A agarose slurry (Millipore). IgG (Santa Cruz and Novus Biologicals) or primary antibody against HIF-1α (Santa Cruz), HIF-2α (Novus Biologicals), or HIF-1β (Novus Biologicals) was incubated with precleared lysates. Salmon sperm DNA/protein A agarose slurry was added and the agarose beads were washed sequentially with: low- and high-salt immune complex wash buffers; LiCl immune complex wash buffer; and twice with TE buffer. DNA was eluted in 1% SDS/0.1 M NaHCO_3_ and crosslinks were reversed by addition of NaCl to 0.2 M. Proteinase K was added to degrade any protein including nucleases. DNA was recovered by phenol-chloroform extraction and ethanol precipitation, treated with RNase, and analyzed by qPCR. Primer sequences are listed in Table S1.

### Luciferase reporter assays

Hep3B cells in 48-well plates were co-transfected with pGL2 firefly luciferase reporter plasmid with HRE-WT or HRE-MUT sequences and pSV-Renilla. 24 h after transfection, cells were exposed to 20% or 1% O_2_ for 24 h and lysed. Luciferase activities were determined with a multi-well luminescence reader (PerkinElmer) using the Dual-Luciferase Reporter Assay System (Promega). Firefly:Renilla luciferase ratio was calculated and normalized to HRE-WT-20% O_2_.

### Xenograft assays

All animal protocols were approved by The Johns Hopkins University Animal Care and Use Committee. Male SCID mice 5 to 7 weeks of age were used. Hep3B cells were resuspended at 2.5 × 10^7^ cells/ml in a 1:1 mix of DMEM:Matrigel (BD Biosciences). A 200-μL suspension containing 5×10^6^ cells was implanted subcutaneously into the right flank. Mice were monitored for body weight and tumor volume (mm^3^), which was calculated as length (mm) × [width (mm)]^2^ × 0.52. When tumor volume reached 200 mm^3^ on day 45, mice were randomly divided into control and treatment groups and received daily intraperitoneal injections of vehicle or 5 mg/kg of GYKI 52466, respectively.

### Immunohistochemistry

Xenograft tumors were fixed in 10% formalin, paraffin embedded, and 5-μm sections were prepared, dewaxed with xylene, hydrated with graded ethanol, followed by antigen retrieval using citrate buffer (pH 6.1). The LSAB+ System HRP kit (DAKO) was used with Ki67 antibody (1:200, Novus Biologicals). Sections were counterstained with Mayer's hematoxylin, dehydrated through graded ethanol into xylene, and mounted. The Ki67 staining was quantified using ImageJ software (NIH) [64].

### Flow cytometry

Cultured cells were washed, detached, and incubated with PE-conjugated Annexin V and 7-amino-actinomycin (7-AAD) in 1X binding buffer (BD Pharmingen) for 15 min at room temperature in the dark. Cell samples were analyzed with a LSR-II flow cytometer (Becton Dickinson).

### Migration and invasion assays

For migration assays, RCC4 and RCC4-VHL cells were seeded onto uncoated inserts of a 24-well Transwell chamber (8-mm pore size; Costar) and allowed to migrate for 16 h in the presence of vehicle, 250 μM GYKI 52466, or 10 μM Saracatinib. For invasion assays, Transwell inserts were coated with Matrigel (BD Biosciences) at 37ºC for 4 h and then cells were seeded onto the inserts. Cells were incubated for 24 h in the presence of vehicle, 250 μM GYKI 52466, or 10 μM Saracatinib. The cells that remained on the upper surface of the filter were removed with a cotton swab. The cells that migrated/invaded to the underside of the inserts were fixed with 100% methanol for 5 min and stained with 0.1% crystal violet for 20 min. Membranes were cut from the Transwell and rinsed in 1 mL of 33% acetic acid. Absorbance was read at 570 nm.

### Correlation analysis of microarray expression data

Gene expression data from Kidney Renal Clear Cell Carcinoma (TCGA) were obtained from the International Cancer Genome Consortium (http://icgc.org/). Pearson's correlation coefficient was used to determine *p* values for co-expression.

### Statistical analysis

Data were analyzed with an unpaired two-tailed Student's *t*-test or ANOVA followed by Bonferroni post-test as indicated in the figure legend. A *p* value < 0.05 was considered significant.

## SUPPLEMENTARY MATERIAL, FIGURES AND TABLE


