# Supplementary material for: Hypoxia-inducible factors enhance glutamate signaling in cancer cells
Source: Oncotarget. 2014 Oct 15;5(19):8853–68. doi: 10.18632/oncotarget.2593 (PMC4253402; doi:10.18632/oncotarget.2593)
Supplement: Supplementary file 1 [file oncotarget-05-8853-s001.pdf]

Hypoxia-inducible factors enhance glutamate signaling in cancer cells

Supplementary Material

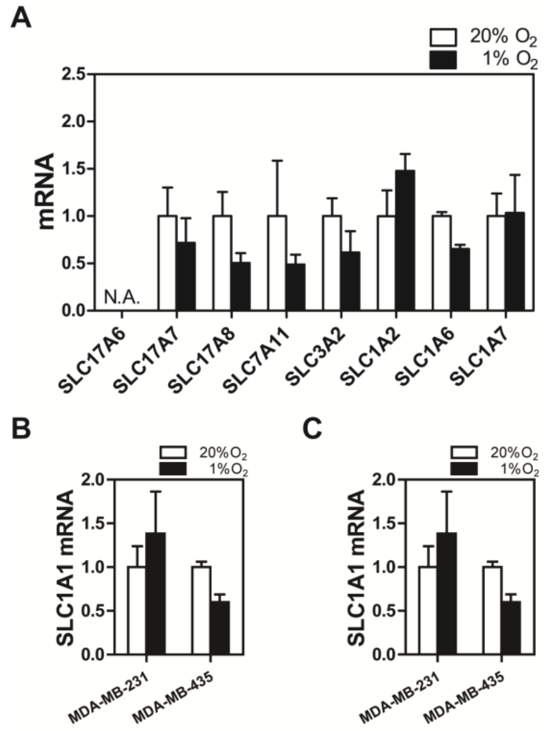

Figure S1: Analysis of glutamate transporter gene expression in Hep3B and breast cancer cells. (A) Hep3B cells were exposed to 20% or 1% O_2 for 24 h. mRNAs were analyzed and normalized to 20% O_2. (B-C) Two breast cancer cell lines, MDA-MB-231 and MDA-MB-435, were exposed to 20% or 1% O_2 for 24 h. *SLC1A1* (B) and *SLC1A3* (C) mRNAs were analyzed and normalized to 20% O_2. Data are mean ± SEM from ≥ 3 experiments.

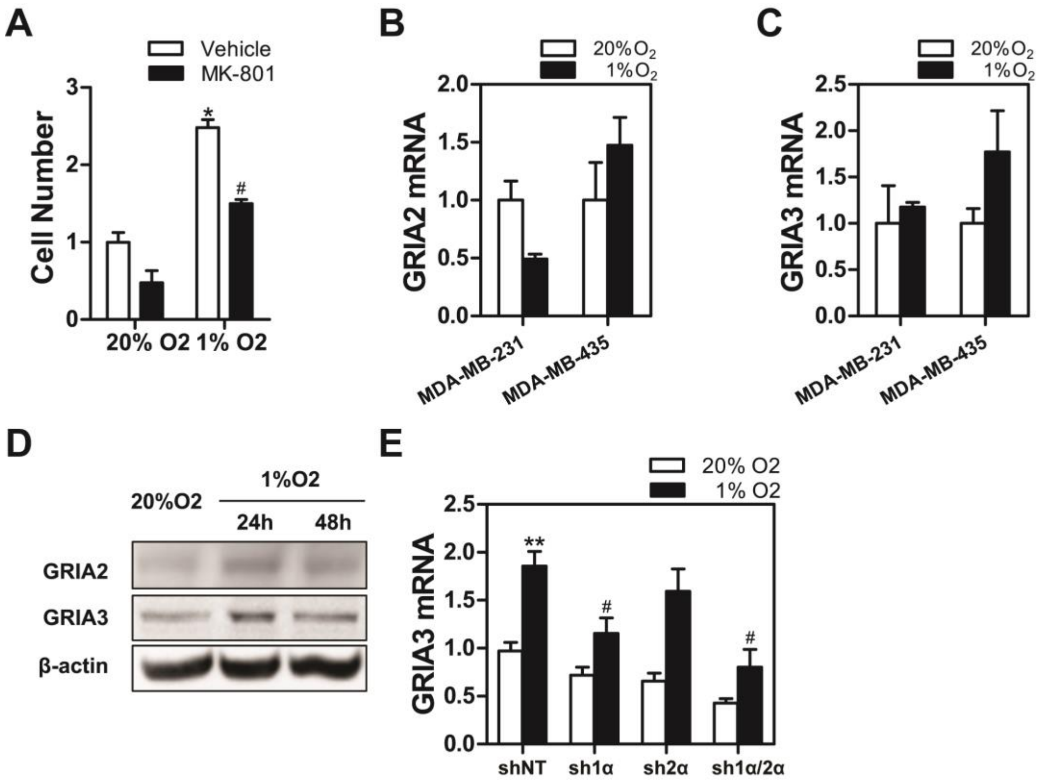

Figure S2: MK-801 inhibits proliferation and hypoxia-induced *GRIA3* expression is HIF dependent in Hep3B cells. (A) Hep3B cells were exposed to 20% or 1% O_2 + vehicle or MK-801 for 96 h and counted. $^{*}P < 0.05$ vs vehicle-20% O_2, $^{\#}P < 0.05$ vs vehicle-1% O_2, ANOVA with Bonferroni post-test. (B-C) MDA-MB-231 and MDA-MB-435 cells were exposed to 20% or 1% O_2 for 24 h. GRIA2 (B) and GRIA3 (C) mRNA was analyzed and normalized to 20% O_2. (D) Hep3B cells were exposed to 20% or 1% O_2. Immunoblot assays were performed using the indicated antibodies. (E) GRIA3 mRNA was analyzed in Hep3B subclones exposed to 20% or 1% O_2 for 24 h. $^{**}P < 0.01$ vs shNT-20% O_2; $^{\#}P < 0.05$ vs shNT-1% O_2, two-way ANOVA with Bonferroni post-test. Data are mean ± SEM or a representative blot from ≥ 3 experiments.

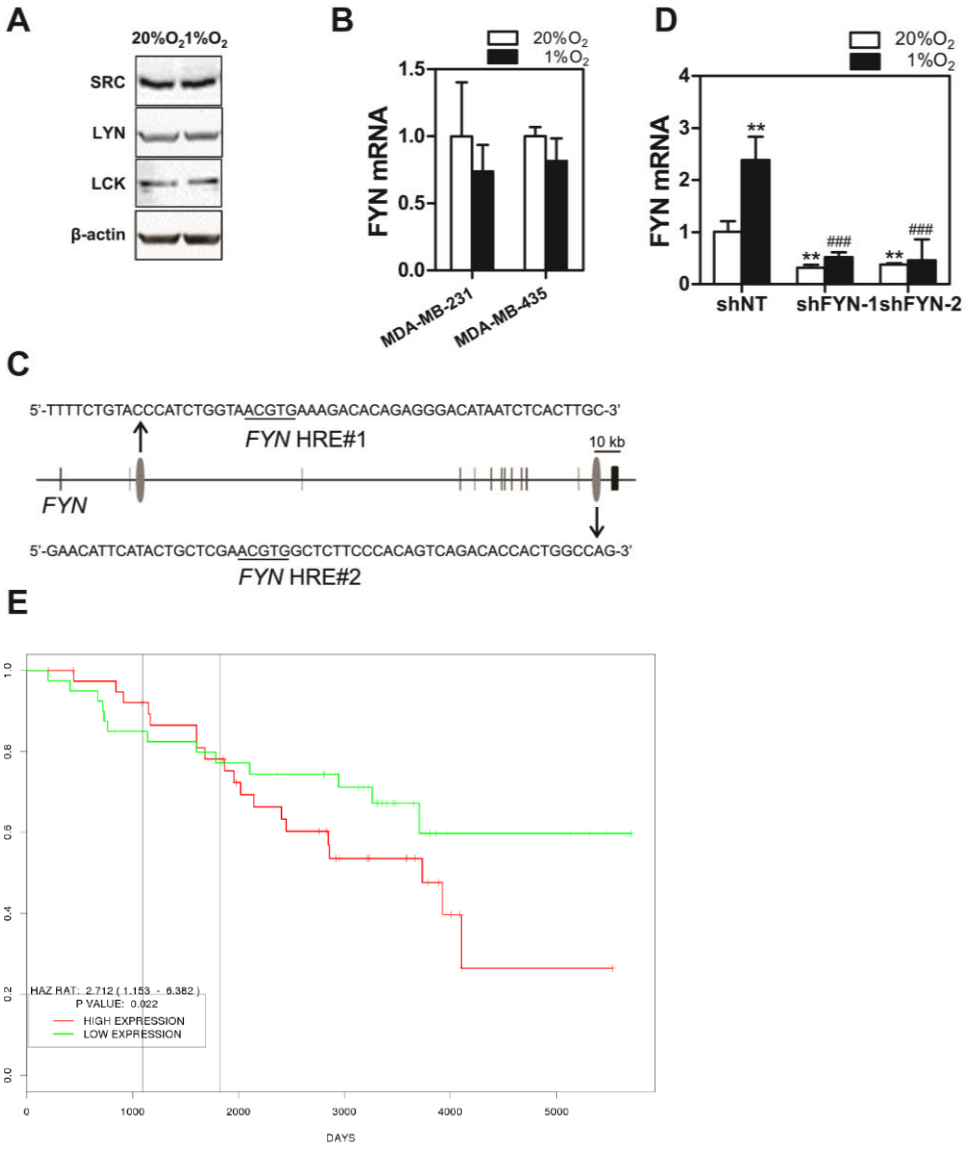

Figure S3: Analysis of SRC family kinase expression in Hep3B cells, *FYN* expression in breast cancer cells, and *FYN* knockdown in Hep3B cells. (A) Hep3B cells were exposed to 20% or 1% O_2 for 24 h. Immunoblot assays were performed using the indicated antibodies. A representative blot from ≥ 3 experiments was shown. (B) MDA-MB-231 and MDA-MB-435 cells were exposed to 20% or 1% O_2 for 24 h. FYN mRNA was analyzed and normalized to 20% O_2. Data are mean ± SEM from ≥ 3 experiments. (C) *FYN* exons and HREs are indicated by black bars and grey oval, respectively; HRE sequences are shown. (D) Hep3B subclones with FYN knockdown were exposed to 20% or 1% O_2 for 24 h. mRNAs were analyzed and normalized to shNT-20% O_2. $^{**}P < 0.01$ vs shNT-20% O_2, $^{\#\#\#}P < 0.001$ vs shNT-1% O_2, ANOVA with Bonferroni post-test. Data are mean ± SEM from ≥ 3 experiments. (E) Kaplan-Meier prognostic plot of overall survival for hepatocellular carcinoma patients stratified by combined gene expression of *SLC1A1*, *SLC1A3*, *GRIA2*, *GRIA3* and *FYN* in data set GSE10141. The two vertical lines indicate three years and five years each. $P = 0.022$.

A

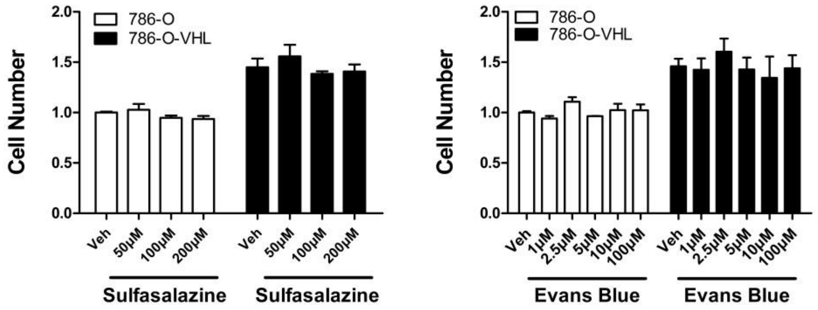

B

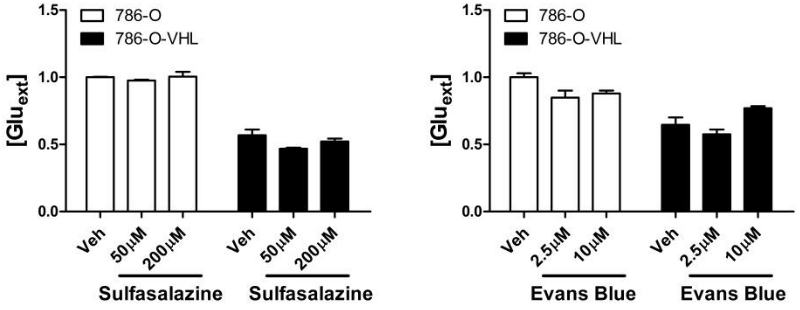

Figure S4: Vesicular glutamate transporters and cystine-glutamate antiporter do not contribute to elevated glutamate efflux by 786-O cells. (A) Cells were treated for 48 h with vehicle (Veh), or with indicated concentration of Sulfasalazine (left panel) or Evans Blue (right panel), and counted. (B) Glutamate concentrations in media were measured and normalized to results for 786-O treated with Veh. Data are mean ± SEM from ≥ 3 experiments.

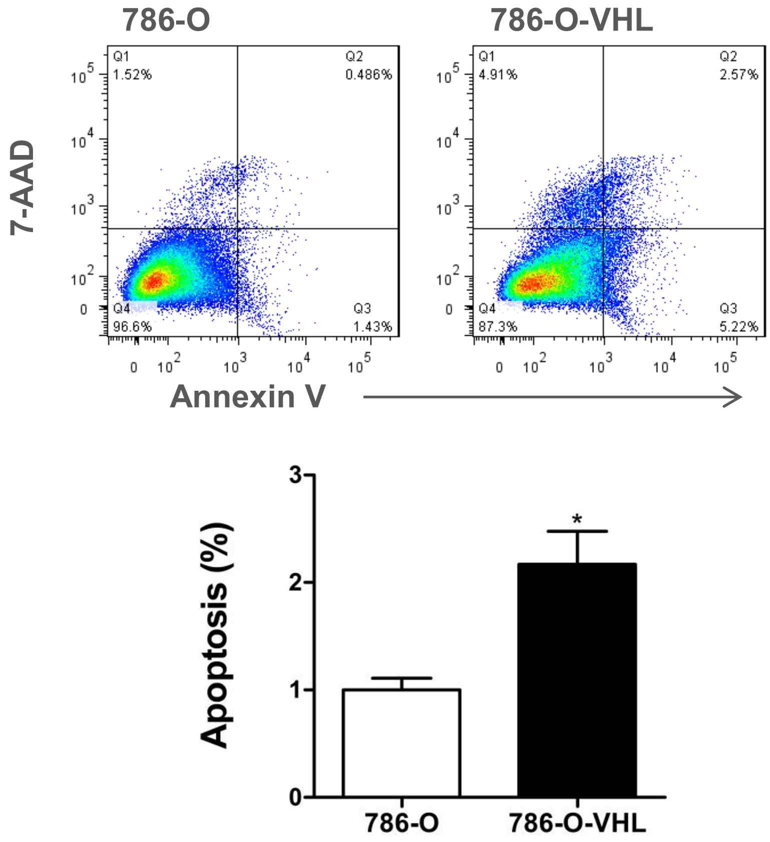

Figure S5: Analysis of apoptosis in 786-O and 786-O-VHL cells. Spontaneous apoptosis in 786-O and 786-O-VHL cells was measured by PE-Annexin V staining by flow cytometry (upper panel). Quadrant 3 (Q3), representing early apoptosis, was quantified and normalized to 786-O (lower panel). $^{*}P < 0.05$ vs 786-O, Student's *t* test. Data are mean ± SEM from ≥ 3 experiments.

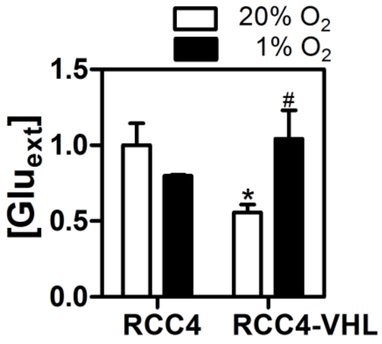

Figure S6: Analysis of glutamate in RCC4 and RCC4-VHL cells exposed to hypoxia. Glutamate concentrations in media were measured and normalized to results for RCC4 at 20% O_2. $^{*}P < 0.05$ vs RCC4-20% O_2, $^{\#}P < 0.05$ vs RCC4-VHL-20% O_2, ANOVA with Bonferroni post-test. Data are mean ± SEM from ≥ 3 experiments.

Table S1: Oligonucleotide sequence of shRNAs, qRT-PCR primers, and ChIP primers.

**shRNAs**	
HIF-1α	CCAGTTATGATTGTGAAGTTA
HIF-2α	GGAGACGGAGGTGTTCTAT
FYN #1	GCCTATTCACTTTCTATCCGT
FYN #2	GTGCCAACAATCCTAGTGCTT
Non-targeting	CAACAAGATGAAGAGCACCAA
**Primers for qRT-PCR assays**	
SLC1A1	Forward: CTTGGAATCCACAATCCTTG
	Reverse: GTGAGGTCTGGGTGAATGAG
SLC1A2	Forward: AGTGCTGGAACTTTGCCTGT
	Reverse: CATCCATGTTAATGGTTGCTC
SLC1A3	Forward: AAACCAAGCGTGAAGAAGTG
	Reverse: AAGATAATCAGGCCCAGGAC
SLC1A6	Forward: ACAGTTCAAGACGCAGTACAG
	Reverse: CCAAGGCCCGAGTGACATTTT
SLC1A7	Forward: CCCGAGGTCGTTTACAAGTCA
	Reverse: GAAGGGGAAATACCACACAGC
SLC7A11	Forward: AGGGTCACCTTCCAGAAATC
	Reverse: GAAGATAAATCAGCCCAGCA
SLC3A2	Forward: CTGGTGCCGTGGTCATAATC
	Reverse: GCTCAGGTAATCGAGACGCC
SLC17A6	Forward: GGGAGACAATCGAGCTGACG
	Reverse: TGCAGCGGATACCGAAGGA
SLC17A7	Forward: CAGAGTTTTCGGCTTTGCTATTG
	Reverse: GCGACTCCGTTCTAAGGGTG
SLC17A8	Forward: CCTCCCCAAGCGTTACATCAT
	Reverse: GCTGTCTGAATTTCCGGTTTTCC
GRIA1	Forward: GGTCTGCCCTGAGAAATCCAG
	Reverse: CTCGCCCTTGTCGTACCAC

GRIA2	Forward: GTGGCTAGAGTGCGGAAGTC
	Reverse: CACCAACTTTCATGGTGTCG
GRIA3	Forward: CGAGAGGGGTGTATGCCATC
	Reverse: GAAGCTAGGCGTAACAAAGGAT
GRIA4	Forward: ATTGGTGTCAGCGTGGTCTTA
	Reverse: CCAGGGAAAACCAGAGGCT
FYN	Forward: CTCAGCACTACCCCAGCTTC
	Reverse: CATCTTCTGTCCGTGCTTCA
18S rRNA	Forward: CGGCGACGACCCATTCGAAC
	Reverse: GAATCGAACCCTGATTCCCCGTC
**Primers for ChIP assays**	
SLC1A3 HRE	Forward: TAATGGAGCTGCCACCCTAT
	Reverse: CAAGCTCCTCCATCTGAAGC
GRIA2 HRE	Forward: CCGAGCTGTGCTTTCTCAG
	Reverse: AGAGAGGGGCAGGCAGTC
FYN HRE#1	Forward: AGCCTATGGCCACAAGTGTT
	Reverse: GAGCTGGGAGCAAGTGAGAT
FYN HRE#2	Forward: TTGGAACAAAATTGGGCAGT
	Reverse: CCAGTGGTGTCTGACTGTGG
